# Active Pitch Stabilization of Tracked Platforms Using a Nonlinear Dynamic Model for Coordinated Inertial Actuation

**DOI:** 10.3390/s26051517

**Published:** 2026-02-27

**Authors:** Alina Fazylova, Kuanysh Alipbayev, Makpal Nogaibayeva, Teodor Iliev, Ivaylo Stoyanov

**Affiliations:** 1 Department of Smart Technologies in Engineering, International Engineering and Technological University, Almaty 050060, Kazakhstan; a.fazylova@aues.kz; 2 Department of Aerospace and Electronic Engineering, Almaty University of Power Engineering and Telecommunications Named after Gumarbek Daukeyev, Almaty 050013, Kazakhstan; k.alipbayev@aues.kz (K.A.); m.nogaibaeva@aues.kz (M.N.); 3 Department of Telecommunications, University of Ruse, 8 Studentska Str., 7004 Ruse, Bulgaria; 4 Department of Electric Power Engineering, University of Ruse, 8 Studentska Str., 7004 Ruse, Bulgaria; stoyanov@uni-ruse.bg

**Keywords:** tracked mobile platform, drive system, inertial actuator, flywheel, body stabilization, coordinated control, nonlinear dynamics, optimal control, mathematical modeling

## Abstract

This study addresses the problem of actively stabilizing the longitudinal body inclination of a tracked mobile platform operating over uneven terrain. A novel drive system architecture is proposed that combines conventional track traction electric drives with an inertial body-stabilization drive based on a flywheel mounted on the pitch axis between the chassis and the body module. The main contribution of the proposed approach is the coordinated control of the traction drives and the inertial actuator based on a unified dynamic model of the platform. A quadratic performance criterion is formulated, and a coordinated optimal control law is synthesized to limit body angular oscillations while accounting for actuator energy consumption. Simulation results for motion over step-like and random terrain irregularities, as well as under external moment disturbances, demonstrate a significant reduction in both peak and root-mean-square pitch-angle deviations relative to configurations without an inertial actuator and with local body stabilization. The results obtained confirm the potential and effectiveness of inertial stabilization drives as part of coordinated drive control systems for tracked mobile platforms intended for special-purpose applications, and indicate prospects for their use in advanced terrestrial robotic platforms and future space robotic systems operating in challenging environments.

## 1. Introduction

Tracked mobile platforms intended for special-purpose applications are widely used in scenarios where body stability requirements are comparable in importance to terrain possibility and traction performance. Such platforms are commonly employed in industrial inspection robotics, infrastructure monitoring, agricultural field automation, environmental surveying, and planetary exploration missions, where reliable operation over uneven terrain, slopes, loose soils, and obstacle-rich environments is required. Under these conditions, dynamic load redistribution between tracks, terrain-induced disturbances, and inertial effects may significantly affect vehicle stability, traction efficiency, and operational accuracy of onboard sensing and technological equipment. From the standpoint of dynamic stability, the key problem addressed in this study is the suppression of longitudinal body pitch oscillations during motion over uneven terrain. These oscillations reduce traction efficiency, affect the accuracy of onboard sensing and technological systems, increase dynamic loads on structural elements, and may lead to degraded operational reliability. Existing stabilization approaches only partially address this problem, underscoring the need for further development of coordinated stabilization strategies. Unlike general-purpose transport vehicles, such platforms are subject to longitudinal angular oscillations of the body, which directly limit the operational effectiveness of optoelectronic systems, stabilized payloads, and onboard measurement equipment. When operating over uneven terrain, the platform body undergoes significant longitudinal and lateral oscillations, which have prompted extensive research on suspension systems, stabilization, and motion control of ground robotic complexes [1]. Existing approaches to reducing body angular oscillations in tracked platforms are predominantly based on optimizing suspension parameters and implementing active or semi-active suspension systems. Various passive and quasi-zero-stiffness suspension designs have been proposed to reduce body accelerations, maintain continuous contact between the tracks and the ground, and mitigate the risk of tip-over when traversing complex terrain [2]. However, such solutions affect the body indirectly—through the vertical dynamics of the chassis—and do not explicitly treat the formation of a controllable pitch-stabilization moment as an independent control objective. In parallel, active and hierarchical control systems have been developed that coordinate suspension behavior and traction drives to improve body stability and reduce energy losses in tires or tracks, particularly during maneuvering and turning motions [3]. Several studies address motion stability through trajectory planning that accounts for terrain geometry and static and dynamic stability criteria [4].

Existing approaches to reducing body oscillations in tracked platforms can be conditionally divided into three main groups: suspension-based stabilization methods, motion planning and traction coordination strategies, and inertial actuator-based stabilization concepts.

While these approaches are effective at the navigation and motion-planning levels, they generally do not incorporate an actuator specifically designed to directly generate a stabilizing moment on the vehicle body during motion. Instead, they primarily rely on terrain geometry and suspension capabilities, without introducing additional actuators dedicated to body stabilization. At the same time, research has advanced the use of inertial actuators—such as flywheels and reaction wheels—for stabilizing the angular orientation of mechanical systems. Reaction wheels are widely employed in spacecraft attitude control, as well as in balancing robots and inverted pendulum systems [5]. It has been shown that inertial actuators can generate stabilizing moments independently of ground contact, which is particularly important under conditions of limited traction and adhesion. More recent studies have explored the use of inertial actuators in legged and hybrid mobile platforms to enhance maneuverability and enable complex maneuvers; however, these solutions are typically focused on relatively lightweight laboratory-scale or small-sized systems [6]. Despite the extensive development of active suspension technologies and the widespread application of inertial actuators in balancing robots, the integration of an inertial body-stabilization drive directly into the drive system of a tracked mobile platform remains underexplored. In most existing studies, the body of a tracked vehicle is treated as a passive element influenced by suspension dynamics and terrain-induced disturbances. At the same time, control moments are generated exclusively through traction drives or suspension components. Moreover, a unified approach to the coordinated control of traction drives and an inertial actuator, based on a common dynamic model that explicitly accounts for the coupling among longitudinal motion, body angular dynamics, and energy consumption, has not yet been established [7]. The objective of this study is to develop a coordinated control approach for an inertial body stabilization drive and the traction drives of a tracked mobile platform based on a unified nonlinear dynamic model. To achieve this objective, the following tasks are addressed. First, a structural configuration for an inertial body stabilization drive, based on a flywheel mounted on the pitch axis between the chassis and the body module, is proposed. Then, using a Lagrangian formulation, a nonlinear dynamic model of the tracked platform motion in the longitudinal plane is derived, incorporating flywheel dynamics and terrain-induced disturbances. Based on the linearization of this model, an optimal coordinated control problem for traction drives and the inertial actuator is formulated, accounting for energy-consumption constraints. Finally, numerical simulations of motion over step-like and random terrain irregularities are performed to evaluate the influence of the inertial actuator on body stabilization performance under representative operating conditions. Contemporary research on the dynamics of tracked and heavy mobile platforms is largely focused on the development of active and semi-active suspension systems to reduce body vibrations and improve ride smoothness when operating over uneven terrain. For tracked vehicles and combat platforms, multi-mass “body–road wheels–track” models are commonly developed, from which active control systems are synthesized using preview controllers, sky-hook and ground-hook schemes, and robust and optimal control algorithms, including model predictive control (MPC). These approaches demonstrate a noticeable improvement in ride comfort, pointing accuracy, and protection of onboard equipment compared with passive suspension systems [8]. At the same time, a classic trade-off between ride comfort and handling performance is emphasized, which is typically mitigated through combined sky- and ground-hook strategies and an integrated design of suspension hardware and control algorithms [9].

In parallel, research has advanced in active anti-roll control, in which control-moment gyroscopes (CMGs) and flywheels are employed as actuators. For heavy road vehicles, articulated loaders, and compact tractors, CMG-based roll-stabilization schemes have been proposed, demonstrating a significant reduction in rollover risk during maneuvers and under external disturbances [10]. However, in all of these studies, active suspension systems and gyroscopic devices were treated as independent actuation subsystems: they either affect the vertical relative motion of the suspension or influence the overall roll dynamics of the vehicle, but do not implement an inertial actuator structurally integrated between the chassis and the body of a tracked platform and treated as a full-fledged element of the unified drive system dynamic model. Moreover, body angular dynamics are typically analyzed under small-angle approximations and without particular emphasis on long-duration operation over strongly disturbed terrain profiles, where body pitch oscillations critically limit the functionality of onboard systems.

Several studies address specific designs of tracked and wheel–track mobile platforms with passive or partially movable suspensions. For instance, in ref. [11], a tracked mobile robot with passive suspension and a differential mechanism between the left and right tracks was proposed; it was shown that this configuration improves track–terrain contact and enhances traversability on rough terrain. However, body posture remains strongly dependent on the terrain profile and is rigidly determined by suspension kinematics, with no means of purposeful pitch stabilization. In ref. [12], an MBD–DEM model of a tracked vehicle equipped with symmetric suspensions was developed, enabling the detailed analysis of dynamic regimes during straight-line motion, climbing, and obstacle negotiation. In that work, the body was modeled as a single rigid body, and the conclusions focused on suspension parameter selection and track–ground interaction, without incorporating a dedicated actuator for active body-orientation control. In ref. [13], a kinematic strategy for a robot with a passively articulated tracked suspension (PASTRo) was proposed, aimed at improving trajectory tracking by accounting for local terrain slopes and distributing velocities between the tracks; nevertheless, body orientation was solely determined by suspension geometry and was not controlled by an independent actuator. Similarly, in ref. [14], a simplified trajectory-tracking strategy was developed for a wheel–track robot with passive suspension, minimizing computational cost and energy consumption; however, purposeful stabilization of the longitudinal body inclination was not addressed as a separate control objective.

Thus, even in studies that explicitly consider multibody dynamics and track–terrain interaction in detail, the body of a tracked platform remains a passive object, and the possibility of introducing an additional inertial actuator between the chassis and the body is not explored.

At the same time, there is an extensive body of research on inertial actuators based on flywheels and reaction wheels, which are widely used to stabilize unstable mechanical systems and control robot orientation. In ref. [15], a discrete inverse optimal control law based on a Lyapunov function was proposed for a classical reaction wheel pendulum, ensuring global stabilization and exponential convergence under bounded actuator torque. In ref. [16], a wheel-based inverted pendulum system was investigated, including controller design, hardware implementation, and state estimation using an LQR regulator and a state observer; the results demonstrated that a reaction wheel could effectively serve as an additional actuator to improve stability and transient response quality. Subsequent studies presented comparative analyses of various control strategies for reaction wheel pendulums—such as sliding-mode control, backstepping, and energy-based methods—thereby confirming the maturity of the theoretical and methodological framework for flywheel-based actuation systems [17].

However, these studies primarily focused on relatively simple laboratory-scale systems consisting of a single rigid body and a single flywheel, without coupling to a tracked chassis. They are typically limited to stabilization around a single operating configuration and do not address prolonged motion over uneven terrain. In particular, inertial actuators are not treated as structural elements between the chassis and the body of a heavy tracked platform, and coordinated control of traction-drive torques and inertial-actuator torque within a unified nonlinear dynamic model is not addressed.

Therefore, despite substantial progress in suspension design and inertial actuator applications, the integration of an inertial stabilization actuator directly into the drive system of a tracked mobile platform, combined with coordinated control of traction drives based on a unified nonlinear dynamic model, remains insufficiently explored. This gap forms the scientific rationale of the present study.

This specific aspect—namely, the integration of an inertial body stabilization actuator into the overall drive system of a tracked mobile platform and the synthesis of coordinated control to reduce pitch oscillations during motion over uneven terrain—remains insufficiently studied. It constitutes the research gap addressed in the present work.

The remainder of this paper is organized as follows. Section 2 introduces the physical system, modeling assumptions, and actuator constraints, and presents the Lagrangian-derived dynamic model together with its transformation into state-space form. The control problem is explicitly formulated, including assumptions on disturbances, measurements, and actuator limitations. Section 3 consolidates the methodological design, including controller synthesis and stability analysis. Section 4 presents the simulation scenarios and results, followed by discussion and concluding remarks.

## 2. Materials and Methods

The object of the present study was a mobile robotic platform with a rigid body mounted on a supporting system (chassis) and intended for operation over rough and uneven terrain. Within the scope of this work, the body was treated as a dynamically decoupled subsystem with its own angular dynamics in the longitudinal plane, namely, pitch. Particular attention was paid to the body’s rotational motion relative to the chassis, since longitudinal angular oscillations are the dominant factor governing dynamic loads on the payload, the accuracy of stabilized subsystems, and the operational reliability of onboard equipment during motion over uneven terrain.

The dynamic model of the tracked platform body was formulated using classical rigid-body rotational dynamics, with suspension effects represented by equivalent torsional stiffness and damping. This approach is widely used in modeling ground robotic platforms and provides an adequate approximation of pitch stabilization dynamics under moderate terrain disturbances.

From the mechanical modeling perspective, the body was treated as an absolutely rigid body connected to the chassis through an equivalent torsional spring–damper element. This element represents the aggregated effect of the suspension components, structural joints, and load-bearing connections between the chassis and the body. Such a representation enables the reduction of a spatial multibody model to an equivalent single-degree-of-freedom rotational model in the pitch plane while preserving the key physical effects relevant to body stabilization.

The pitch axis passes through the attachment point between the body and the chassis and is oriented perpendicular to the platform’s plane of longitudinal motion. The body’s center of mass is located a certain distance from this axis, resulting in a gravitational restoring torque when the body is deflected from its equilibrium position. In the following analysis, the body’s pitch angle was adopted as the sole generalized coordinate, thereby enabling a consistent formulation of the system’s Lagrangian and the proper incorporation of both gravitational and inertial effects.

**Figure 1:**
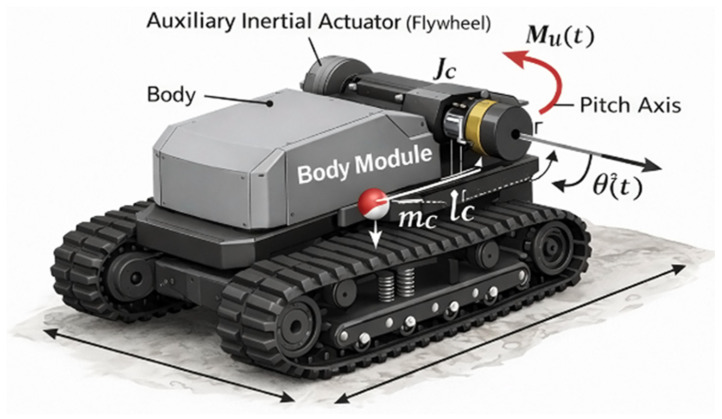
3D representation of the tracked mobile platform with an integrated inertial stabilization actuator and indication of the main physical parameters used in the dynamic model.

Figure 1 illustrates the tracked chassis, body module, auxiliary inertial actuator (flywheel), pitch rotation axis, center of mass, and principal geometric dimensions. Symbolic designations correspond to the parameters introduced in the mathematical formulation: *θ*(*t*)—body pitch angle; m_c_—body mass; *J_c_*—body moment of inertia relative to the pitch axis; *l_c_*—distance from the pitch axis to the center of mass; *M_u_*(*t*)—control torque generated by the inertial actuator; *M_r_*(*t*)—disturbance torque caused by terrain interaction; *k_θ_* and *d*—equivalent torsional stiffness and damping coefficients. The model is primarily valid in the small-angle regime (typically ±5–10°), whereas nonlinear terms are retained to capture transient and limiting dynamic conditions accurately. The 3D visualization provides a physical interpretation of the simplified analytical model and clarifies the relationship between the platform geometry, mass distribution, and the adopted stabilization dynamics.

The geometric configuration, mass distribution, and inertial properties used in the mathematical model were determined from a three-dimensional CAD representation of the platform prototype. Figure 1, therefore, combines physical visualization with symbolic parameter indication, ensuring consistency between the real system configuration, the adopted dynamic abstraction, and the simulation assumptions. Such representation improves the interpretability of the stabilization model and facilitates the reproducibility of the reported results.

The numerical values, typical ranges, and explanatory notes for the model parameters used in simulations are summarized in Table 1.

**Figure 2:**
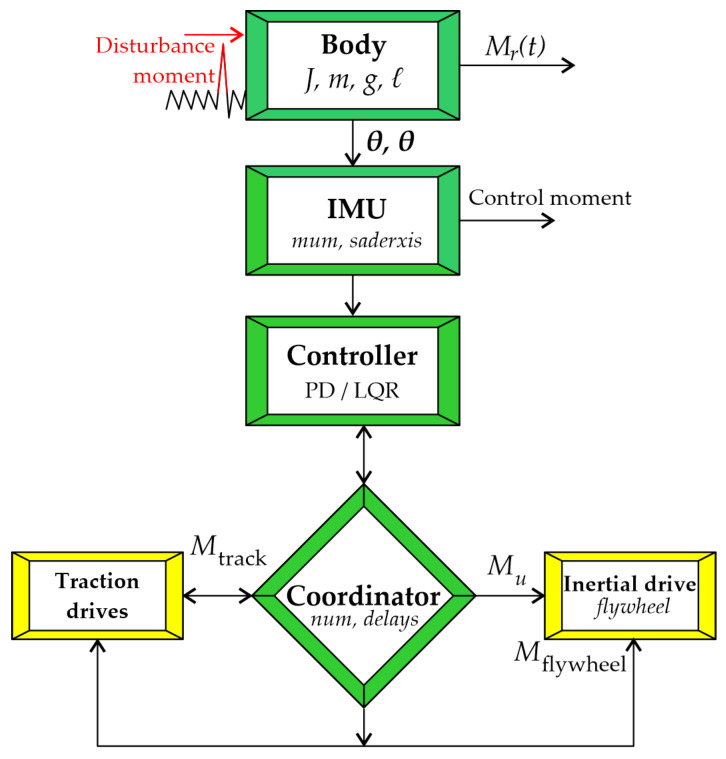
Structural diagram of the coordinated drive control system of the tracked mobile platform.

To provide a clear representation of the relationships between the physical body model, the measurement system, the control algorithms, and the actuators, a structural block diagram of the coordinated control system was employed, as shown in Figure 2. The color coding in Figure 2 serves to separate the physical plant components from the control and signal-processing layers. Yellow blocks denote hardware subsystems (mechanical body, traction drives, inertial actuator), while the internal white fields describe their associated dynamic parameters and embedded functions. The coordination block is emphasized by its diamond shape to indicate its supervisory role in torque distribution. Red arrows indicate disturbance inputs, and black arrows represent measurement and control signal flow within the closed-loop architecture. This diagram reflects the architecture of control torque generation. It serves as a logical foundation for the subsequent description of body angle and angular velocity measurement methods, as well as for the synthesis of control laws.

The body’s geometric and mass–inertial parameters, including the center of mass and the moment of inertia about the pitch axis, were determined from a three-dimensional CAD model of the platform. These parameters were subsequently used to develop the mathematical model and conduct numerical simulations.

The body’s mass–inertial characteristics are key parameters governing pitch dynamics and the formulation of the mathematical model of the stabilization system. According to the geometric model shown in Figure 1, the body was treated as an absolutely rigid body rotating about the pitch axis passing through its attachment point to the chassis. The principal parameters determining the body dynamics in the pitch plane are the body mass mc, the location of the center of mass relative to the rotation axis, and the moment of inertia *J_c_* about the pitch axis. The total body mass *m_c_* was determined as the sum of the masses of all structural and functional components rigidly attached to the platform body, including the load-bearing structure, power elements, onboard equipment, power supply units, and the payload in its nominal configuration. The mass value was taken as [18]:

(1) $$m_{c}=220\;\mathrm{kg},$$

which corresponds to the platform prototype’s design specifications and was corroborated by the three-dimensional CAD model and the technical specifications of the employed components [19]. The use of the design-based mass value enables the considered scenarios to reflect realistic operating conditions closely and eliminates the need for artificial parameter tuning of the dynamic model.

The position of the body’s center of mass was determined from a three-dimensional geometric model of the platform, accounting for the mass distribution of all components. For further analysis, the projection of the distance from the pitch axis to the center of mass along the vertical direction was used and denoted as *l_c_*. In the baseline configuration, the following value was obtained: *l_c_* = 0.30 m. The nonzero value of lc gives rise to a gravitational restoring moment that acts when the body deviates from the horizontal position and plays a key role in determining the system’s natural frequency and the stability of the passive configuration. The moment of inertia of the body *J_c_* relative to the pitch axis was determined using the CAD model by integrating the mass distribution with respect to the corresponding axis of rotation. To verify the correctness of the obtained value, an equivalent estimation based on the radius of gyration was used [20]:

(2) $$J_{c}=m_{c}k^{2},$$

where *k* is the radius of gyration of the body.

Equation (3) represents the rotational equation of motion of the platform body about the pitch axis, where inertial, damping, stiffness, control, and disturbance torques are taken into account:

(3) $$J_{c}=52.8\;{\mathrm{kg}\times m}^{2},k\approx \sqrt{\frac{J_{c}}{m_{c}}}\approx 0.49\;m.$$

The obtained value of the radius of gyration is comparable to the characteristic geometric dimensions of the body, which confirms the physical correctness of the inertial model. Based on the determined mass and the position of the center of mass, the gravitational moment coefficient is introduced:

(4) $$m_{c}gl_{c}=220\cdot 9.81\cdot 0.30\approx 647\;N\times m.$$

This parameter determines the gravitational field’s contribution to the body’s restoring action and directly affects the system’s dynamic characteristics, including the natural oscillation frequency and stability in the passive mode. The mass–inertial parameters determined in this subsection are used:

- In the formulation of the kinetic and potential energy of the system;
- In the equations of motion of the body in the pitch plane;
- In the calculation of the natural frequency of the system and the selection of quasi-resonant excitation regimes;
- In numerical scenarios related to mass variation, damping degradation, and actuator failures.

Fixing the mass–inertial parameters based on the geometric model ensures the reproducibility of simulation results and eliminates the need to tune coefficients for specific scenarios.

Next, the development of an equivalent elasticity-damping model for the body–chassis connection is considered, along with the methodology for determining the torsional stiffness *k_θ_* and the damping coefficient d used in the mathematical model. The dynamic interaction between the mobile platform body and the supporting system (chassis) is achieved through a set of structural elements, including suspension components, connecting joints, and damping elements. For the purposes of mathematical modeling and numerical analysis, this set was approximated by an equivalent torsional elastic–damping connection in the pitch plane.

This approach is standard in the analysis of transport and robotic system dynamics and allows for reducing a complex spatial problem to a tractable model with physically interpretable parameters. The equivalent torsional stiffness *k_θ_* characterizes the resistance of the system to body deviation from the equilibrium position in the pitch plane. Within the adopted model, the model is formed by the vertical stiffness of the suspension elements and their geometric arrangement relative to the pitch axis. For small deflection angles, the relationship between the vertical displacements of the supports and the pitch angle can be approximated by a linear dependence, which makes it possible to express the equivalent torsional stiffness in the form [21]:

(5) $$k_{\theta }\approx k_{z}\;b^{2},$$

where *k_z_* is the total vertical stiffness of the supporting elements, and b is the characteristic lever arm of force application relative to the pitch axis.

Based on data on the suspension characteristics and the platform geometry, the equivalent stiffness was estimated as *k_θ_* = 800 “N×m/rad”.

This value ensures correct reproduction of the observed body stiffness under static and dynamic loads and corresponds to the range of parameters characteristic of platforms of a similar class. The viscous damping *d* reflects the total energy losses in the system caused by:

- Internal damping of suspension elements,
- Friction in joints and connections,
- Dissipative properties of materials.

Equations (6) and (7) describe the equivalent torsional stiffness and damping contributions reflecting suspension compliance and structural energy dissipation. In the equivalent model, damping is introduced in the form of a linear viscous term proportional to the angular velocity of the body:

(6) $$M_{d}=d\;\dot{\theta }.$$

Similarly to the stiffness, the damping coefficient can be estimated through the vertical damping of the supporting elements:

(7) $$d\approx c_{z}\;b^{2},$$

where *c_z_* is the total vertical damping coefficient.

Within the framework of this study, the following value was used:

(8) d=15 N×m×s/rad,

which provides moderate damping of body oscillations in the passive configuration and corresponds to the observed characteristics of real suspension systems. The combined influence of the stiffness *k_θ_*, damping *d*, and the gravitational moment *m_c_ gl_c_* determines the inherent dynamic characteristics of the passive system. For the linearized model, the equation of motion takes the form [21]:

(9) $$J_{c}\ddot{\theta }+d\dot{\theta }+(k_{\theta }+m_{c}gl_{c})\theta =M_{ext}(t),$$

Equations (10)–(12) define the actuator torque generation and limitation mechanisms, including saturation and rate constraints typical for inertial stabilization systems [22].

(10) $$\omega_{0}=\sqrt{\frac{k_{\theta }+m_{c}gl_{c}}{J_{c}}}.$$

Substitution of numerical values results in a natural frequency value on the order of 0.8–0.9 Hz, which is subsequently used in the formation of quasi-resonant excitation scenarios. The determined stiffness and damping parameters are used:

- In the baseline passive model (Scenarios 1–10 of the Section 4);
- In the analysis of parametric uncertainty (Scenario 4 of the Section 4);
- In modeling damping degradation due to wear (Scenario 7 of the Section 4);
- Under combined adverse conditions (Scenario 9 of the Section 4).

Fixing these parameters based on physical considerations eliminates the need for artificial tuning of the model to individual scenarios and increases the reliability of the results. Next, the structure of the actuators for active stabilization is described, and constraints on the control inputs used in the mathematical model and numerical experiments are introduced.

Active stabilization of the mobile platform body is achieved using actuators that generate controlled moments about the pitch axis. Within the framework of this study, three types of actuation, corresponding to different stabilization system architectures, were considered: force actuation via the support, an inertial actuator (flywheel), and control of the center-of-mass position.

Primary attention was given to the physical limitations of the actuators, since they determine the system’s achievable dynamic characteristics and significantly affect the stability and robustness of control under real operating conditions. In the baseline active configuration, the control action is expressed as a moment u(t) applied about the support contour (suspension or actuator at the body–chassis attachment). Such a moment can be realized, for example, by means of:

- An electromechanical actuator with a gearbox,
- A hydraulic actuator,
- An active suspension element.

Taking into account the characteristics of typical actuators for mobile robotic platforms, the admissible range of the control moment is limited by the value:

(11) $$|u(t)|\leq u_{max},u_{max}=200\;N\times m.$$

This value corresponds to the torque that can be generated by the actuator without overloading the power electronics and mechanical components. In addition to the amplitude limitation, the actuators have a finite rate of change of output torque, which is constrained by the actuator inertia and the power-electronics dynamics. To account for this effect, the following constraint is introduced:

(12) $$|\frac{du(t)}{dt}|\leq {\dot{u}}_{max},{\dot{u}}_{max}=500-600\;N\times m/s.$$

This constraint plays a key role in scenarios involving impulsive disturbances and abrupt transient processes (Scenarios 5, 8, and 9 of the Section 4), where instantaneous generation of the control torque is physically impossible. To extend the functional capabilities of the selective stabilization, an inertial actuator in the form of a flywheel installed inside the body was also considered in the model. The flywheel generates the control torque *T_f_*(t) by varying its own angular velocity. The moment of inertia of the flywheel is determined based on its geometric dimensions and mass and is taken as:

(13) $$I_{f}=0.405\;{\mathrm{kg}\times m}^{2}.$$

Equations (14)–(17) characterize the disturbance torque model and its effect on pitch dynamics during uneven-terrain traversal. The capabilities of the electric drive limit the control torque of the flywheel:

(14) $$|T_{f}(t)|\leq T_{f,max},T_{f,max}=120\;N\times m.$$

The inertial actuator is particularly effective for suppressing high-frequency oscillations and is used in a hybrid scheme jointly with the force control channel. In an alternative architecture of active stabilization, the control action is implemented by shifting the body’s center of mass relative to the pitch axis. Such a shift can be achieved by means of a movable mass installed inside the body. The maximum allowable displacement of the center of mass is limited by the structural dimensions of the mechanism and, in this study, was taken as:

(15) $$|\Delta l(t)|\leq \Delta l_{max},\Delta l_{max}=0.05\;m.$$

The following expression determines the equivalent controllable torque in this case:

(16) $$u_{cm}(t)=m_{c}g\;\Delta l(t)sin\theta (t).$$

It should be noted that the effectiveness of this control channel significantly depends on the magnitude of the angle θ, which limits its applicability in small-deviation regimes. To analyze the robustness of the active system, degradation and partial failure modes of the actuators were introduced into the mathematical model, including:

- A reduction in the maximum allowable torque *u_max_* (Scenario 8 of the Section 4);
- Operation in saturation mode and prolonged rate limitation (Scenarios 5 and 9 of the Section 4).

Such a formulation enables the evaluation of system behavior under fault conditions and confirms the active control system’s ability to ensure graceful performance degradation. The actuators and constraints described in this subsection were used:

- In forming the right-hand sides of the differential equations of motion;
- In active stabilization control algorithms;
- In constructing Scenarios 5–9 of the Section 4, reflecting realistic operating conditions.

Thus, the adopted constraints ensure the physical realizability of the simulation results and exclude nonphysical control regimes. Next, the measurement model, sensors, noise, and delays used in the robustness analysis of the active stabilization system were considered. The effectiveness of the active stabilization system under real operating conditions is largely determined by the quality of state measurements and the control loop’s dynamic properties. In practical systems, measurements of the body pitch angle and angular velocity are inevitably noisy, and the generation of control actions is subject to finite delays arising from signal processing, control-law computation, and actuator dynamics.

Measurement of the pitch angle *θ*(*t*) and angular velocity $\dot{\theta }(t)$ was assumed to be performed using inertial measurement units (IMUs) comprising gyroscopes and accelerometers. Such sensors are characterized by additive noise, which, to a first approximation, can be modeled as a Gaussian random process. The measured values were modeled as follows [22]:

(17) $$\begin{array}{cc} \theta_{m}(t) & =\theta (t)+n_{\theta }(t),\\ {\dot{\theta }}_{m}(t) & =\dot{\theta }(t)+n_{\dot{\theta }}(t), \end{array}$$

where $n_{\theta }\left(t\right)$ and $n_{\dot{\theta }}(t)$ are independent stationary Gaussian noises with zero mean.

Based on the characteristics of typical mid-grade inertial sensors, the standard deviations of the noises were taken as:

(18) $$\sigma_{\theta }=0.5-0.6^{{}^{\circ}},\sigma_{\dot{\theta }}=1.0-1.2^{{}^{\circ}}$$

The selected values correspond to the actual operating characteristics of IMUs without the use of complex high-dimensional filters and reflect a conservative assessment of measurement quality. In the active stabilization system, the control action is not generated instantaneously but with a certain delay, which includes:

- Sensor data sampling and processing time;
- Computation of the control law;
- Generation of the control signal and the actuator response.

Equation (19) summarizes the closed-loop dynamic model used for subsequent simulation studies [22]:

(19) $$u(t)=u_{des}(t-\tau_{d}),$$

where Δ*t* is the numerical integration step.

Such an approach makes it possible to correctly account for the effect of delay without introducing additional approximations into the differential equations of motion. The presence of measurement noise and time delays leads to:

- A reduction in the effective damping generated by active control;
- The appearance of high-frequency fluctuations in the control torque;
- A decrease in the system stability margin.

Accounting for these effects is fundamental to robustness analysis, as they often determine the boundary between stable and unstable behavior in real control systems. To analyze the dynamics of the mobile platform body and evaluate the effectiveness of passive and active stabilization systems, numerical integration of the nonlinear differential equations obtained in the Section 3 was employed. Numerical experiments were conducted under conditions corresponding to various scenarios of external disturbances and operating modes. The system of differential equations describing the motion of the body and the actuators was solved using a step-by-step time-domain integration. A base integration step of Δ*t* = 1 ms was used, which provides sufficient approximation accuracy in the presence of:

- Fast transient processes;
- High-frequency components of the control torque;
- Stochastic disturbances and measurement noise.

The choice of the integration step was motivated by the need to accurately account for rate-of-change constraints on control actions and control-loop delays. Convergence testing with a reduced integration step showed no qualitative changes in the system dynamics.

Based on the adopted modeling assumptions and actuator constraints, the stabilization problem considered in this work consisted of suppressing pitch oscillations of the robotic platform under external disturbances while maintaining energy-efficient operation of the coordinated inertial actuation system. The nonlinear system dynamics derived using the Lagrangian formalism are represented in state-space form, with the pitch angle and angular velocity as state variables, and the control input is generated via coordinated flywheel torque and center-of-mass actuation. External disturbances caused by terrain excitation were assumed to be bounded, measurements were obtained from onboard inertial sensors with limited noise, and actuator torque and rate limits were explicitly taken into account. These assumptions define the control problem addressed in the following section.

## 3. Mathematical Modeling

Building upon the dynamic model and control problem formulation presented in Section 2, this section focuses on controller synthesis and stability analysis for coordinated stabilization of the robotic platform.

The dynamics of the mobile platform body in the plane of longitudinal inclination (pitch) were considered. The pitch angle of the body *θ*(*t*) relative to the support base (“chassis–ground”) was chosen as the single generalized coordinate. It was assumed that:

- The angles in the operating regime were small (|θ| ≤ 20–30°), however, nonlinear terms sinθ were retained in the model for the correct description of limiting regimes;
- The elastic properties of the body–chassis connection were approximated by an equivalent torsional stiffness *k_θ_* and damping *d*;
- The terrain influence was introduced as an external disturbance moment function *M_ext_*(*t*), obtained from the selected scenario (impulses, harmonic excitation, colored noise, etc.).

Thus, the problem reduces to analyzing the stability and suppression of oscillations in θ(t) across various realizations of *M_ext_*(*t*) and different variants of control action formation.

The model equations are introduced below and referenced by their equation numbers for clarity and reproducibility.

The kinetic energy of the rotational motion of the body relative to the hinge is defined as Equation (20) [23]:

(20) $$T=\frac{1}{2}J_{c}{\dot{\theta }}^{2},$$

where *J_c_* is the moment of inertia of the body relative to the pitch axis.

The potential energy consists of the elastic energy of the equivalent torsional element and the gravitational energy due to the displacement of the center of mass by a distance *l*_*c*_ from the rotation. It is written as Equation (21) [24]:

(21) $$V=\frac{1}{2}k_{\theta }\theta^{2}+m_{c}gl_{c}\;(1-cos\;\theta ),$$

where *m_c_* is the body mass, *g* is the acceleration due to gravity, and, *l_c_* is the lever arm of the center of mass.

Rayleigh dissipation function (viscous damping) [25] is given by Equation (22):

(22) $$R=\frac{1}{2}d{\dot{\theta }}^{2}.$$

Using the Lagrange equation of the second kind in Equation (23):

(23) $$\frac{d}{dt}\left(\frac{\partial T}{\partial \dot{\theta }}\right)-\frac{\partial T}{\partial \theta }+\frac{\partial V}{\partial \theta }+\frac{\partial R}{\partial \dot{\theta }}=Q_{\theta },$$

The resulting nonlinear pitch equation is obtained in Equation (24):

(24) $$J_{c}\ddot{\theta }+d\dot{\theta }+k_{\theta }\theta +m_{c}gl_{c}sin\;\theta =Q_{\theta },$$

where *Q_θ_* is the total generalized moment, including the external disturbance and possible control actions.

For small angles, the model can be linearized, resulting in Equation (25):

(25) $$J_{c}\ddot{\theta }+d\dot{\theta }+(k_{\theta }+m_{c}gl_{c})\theta =Q_{\theta }.$$

This form is used for analytical evaluations, selection of characteristic frequencies, and construction of quasi-resonant scenarios. In the passive system, the control action is absent, and the generalized moment is determined only by the terrain (see Equation (26)):

(26) $$Q_{\theta }=M_{r}(t).$$

In summary, Equation (27):

(27) $$J_{c}\ddot{\theta }+d\dot{\theta }+k_{\theta }\theta +m_{c}gl_{c}sin\;\theta =M_{r}(t).$$

Control is implemented according to a feedback law (PD structure), given in Equation (28):

(28) $$Q_{\theta }=M_{r}(t)+u(t),$$

and the equation of motion takes the form (Equation (29)):

(29) $$J_{c}\ddot{\theta }+d\dot{\theta }+k_{\theta }\theta +m_{c}gl_{c}sin\;\theta =M_{r}(t)+u(t).$$

Control is implemented according to a feedback law (a PD structure was used in the scenarios) (Equation (30)):

(30) $$u_{des}(t)=-K_{p}\;\theta_{m}(t)-K_{d}\;{\dot{\theta }}_{m}(t),$$

where $\theta_{m},{\dot{\theta }}_{m}$ measure the (possibly noisy/delayed) quantities.

The actuator amplitude and rate constraints considered in the simulations are formally introduced in Equations (31) and (32):

- Amplitude saturation:

(31) $$|u(t)|\leq u_{max},u(t)=sat(u_{des}(t),u_{max}),$$

- Rate-of-change limitation

(32) $$|\frac{du}{dt}|\leq {\dot{u}}_{max}.$$

This model corresponds to the conventional force-based stabilization channel and most effectively suppresses low- and mid-frequency body oscillations. In addition to torque-based stabilization, an alternative mechanism involves modulating the body’s center of mass, thereby producing an equivalent controllable gravitational torque. Such approaches are commonly used in the rigid-body pitch-dynamics modeling of mobile robotic platforms and inertial stabilization systems [26]. The center-of-mass shift is therefore represented in compact form as:

(33) $$l_{c}(t)=l_{c0}+\Delta l(t),|\Delta l(t)|\leq \Delta l_{max}.$$

In the hybrid stabilization architecture, two coordinated control channels are considered: the support torque *u*(*t*) and an inertial actuator (flywheel) generating torque *T_f_*(*t*). The relative angular position of the flywheel within the body is denoted by *φ*(*t*), and the absolute angular position is defined as:

(34) ψ(t)=θ(t)+φ(t).

The corresponding kinetic energy of the inertial actuator is expressed as:

(35) $$T_{f}=\frac{1}{2}I_{f}(\dot{\theta }+\dot{\varphi }{)}^{2},$$

where *I_f_* is the flywheel moment of inertia.

Detailed intermediate algebraic steps leading to the final coupled equations are omitted for conciseness, since similar rigid-body formulations are well-established in the literature [27]. Taking into account the flywheel-generated control torque, the resulting coupled nonlinear equations of motion used in the simulations are presented directly in Equation (36).

(36) $$\left\{\begin{array}{c} J_{c}\ddot{\theta }+d\dot{\theta }+k_{\theta }\theta +m_{c}gl_{c}\sin\theta =M_{r}(t)+u(t)-T_{f}(t),\\ I_{f}(\ddot{\theta }+\ddot{\varphi })=T_{f}(t). \end{array}\right.$$

(37) $$\ddot{\varphi }=\frac{T_{f}(t)}{I_{f}}-\ddot{\theta }.$$

This coupled model forms the basis for the coordinated stabilization scenarios analyzed in the following section.

To obtain an explicit state-space form suitable for control design and numerical simulations, we can rewrite the nonlinear rotational dynamics in first-order form. Let the state vector be defined as:

(38) $$x(t)=\left[\begin{array}{c} x_{1}(t)\\ x_{2}(t) \end{array}\right]=\left[\begin{array}{c} \theta (t)\\ \dot{\theta }(t) \end{array}\right]$$

Then, by definition:

(39) $${\dot{x}}_{1}(t)=x_{2}(t).$$

Starting from the nonlinear equation of motion derived in Section 3, the pitch acceleration $\ddot{\theta }(t)$ is expressed explicitly by isolating the highest derivative term. Using the adopted notation, the resulting second-order model can be written in the form:

(40) $$J_{c}\;\ddot{\theta }\left(t\right)=-d\;\dot{\theta }\left(t\right)-k_{\theta }\;\theta \left(t\right)-m_{c}gl_{c}sin\;\theta (t)+M_{r}(t)+u(t)-T_{f}(t),$$

which yields:

(41) $${\dot{x}}_{2}(t)=\ddot{\theta }(t)=\frac{1}{J_{c}}(-d\;x_{2}(t)-k_{\theta }\;x_{1}(t)-m_{c}gl_{c}sin\;x_{1}(t)+M_{r}(t)+u(t)-T_{f}(t)).$$

Therefore, the nonlinear state-space representation is obtained as:

(42) $$\dot{x}(t)=f(x(t))+g\;u(t)+w(t),$$

where f(⋅) collects the autonomous dynamics terms, $g={\left[\begin{array}{cc} 0 & 1/J_{c} \end{array}\right]}^{⊤}$, and w(t) represents the disturbance and additional actuation terms (e.g., $M_{r}(t)$ and $T_{f}(t)$).

For cases where the inertial actuator (flywheel) dynamics are included explicitly, the state vector is extended to:

(43) $$x(t)=\left[\begin{array}{c} \theta (t)\\ \dot{\theta }(t)\\ \varphi (t)\\ \dot{\varphi }(t) \end{array}\right],$$

and the coupled equations given in Equation (39) can be rewritten in first-order form by solving the system for $\ddot{\theta }(t)$ and $\ddot{\varphi }(t)$, which directly provides the corresponding state-space model $\dot{x}(t)=F(x(t),u(t),M_{r}(t))$.

To provide theoretical support for the proposed coordinated stabilization approach, we briefly analyze the stability of the closed-loop nonlinear pitch dynamics using a Lyapunov framework commonly adopted in nonlinear robotic control and optimization-based feedback systems [28].

Considering the pitch dynamics in compact form:

(44) $$J_{c}\ddot{\theta }+d\dot{\theta }+k_{\theta }\theta +m_{c}gl_{c}sin\;\theta =M_{r}(t)+u(t)-T_{f}(t),$$

we introduce the nominal coordinated control law:

(45) $$u(t)-T_{f}(t)=-k_{p}\theta -k_{d}\dot{\theta },$$

where $k_{p}>0$, $k_{d}>0$.

A Lyapunov candidate function based on total mechanical energy is chosen as:

(46) $$V(\theta ,\dot{\theta })=\frac{1}{2}J_{c}{\dot{\theta }}^{2}+\frac{1}{2}k_{\theta }\theta^{2}+m_{c}gl_{c}(1-cos\;\theta ).$$

Taking the time derivative and substituting (44) and (45) yields:

(47) $$\dot{V}=-(d+k_{d}){\dot{\theta }}^{2}-k_{p}\theta \dot{\theta }+\dot{\theta }M_{r}(t).$$

In the nominal disturbance-free case $M_{r}(t)=0$, the derivative $\dot{V}\leq 0$, ensuring asymptotic stability of the equilibrium $\left(\theta ,\dot{\theta })=(0,0\right)$ according to LaSalle’s invariance principle.

For bounded disturbances $M_{r}(t)$, the system exhibits ultimate boundedness, consistent with robustness analyses commonly reported in nonlinear cooperative control and optimization-based stabilization studies [29].

## 4. Results and Discussion

This section presents the results of numerical simulations obtained using the proposed dynamic model and control architectures. The analysis focuses on the longitudinal pitch dynamics of the tracked mobile platform body and provides a comparative assessment of passive stabilization and several active stabilization strategies, including force-based, inertial, and hybrid control schemes.

The simulation study was organized into a set of representative scenarios covering deterministic and stochastic terrain excitations, near-resonant conditions, parametric uncertainty, actuator limitations, measurement imperfections, and fault-related regimes. For each scenario, time-domain responses of the body pitch angle were analyzed to evaluate stabilization effectiveness, robustness, and sensitivity to non-ideal operating conditions.

Table 1 summarizes the physical parameters used in the dynamic model and numerical simulations, including typical ranges and explanatory notes to improve reproducibility.

The parameter values were selected based on CAD-derived geometric properties, typical characteristics of medium-scale tracked robotic platforms, and actuator performance specifications reported in related experimental studies. The adopted ranges reflect realistic operating conditions and ensure physical consistency of the simulations.

For the correct selection of external excitation regimes and interpretation of the numerical simulation results, the natural frequency of longitudinal angular oscillations of the body in the vicinity of the equilibrium position is preliminarily determined. This parameter defines the characteristic time scale of the system dynamics and is used in the formation of quasi-resonant scenarios.

In the linear approximation of the equations of motion, the natural angular frequency of the system is determined by the expression:

(48) $$\omega_{0}=\sqrt{\frac{k_{\theta }+m_{c}gl_{c0}}{J_{c}}}=\sqrt{\frac{800+647}{52.8}}\approx 5.24\;\mathrm{rad}/s,$$

which corresponds to the natural frequency:

(49) $$f_{0}=\frac{\omega_{0}}{2\pi }\approx 0.83\;\mathrm{Hz}.$$

The obtained value shows that the pitch dynamics of the body belong to the low-frequency range characteristic of heavy tracked platforms. In the following sections, quasi-resonant regimes are understood as external excitations whose frequency components are located near *M_cmd_*(*t*). Analysis of such regimes makes it possible to evaluate the worst-case amplitude responses of the system and to objectively compare the effectiveness of passive and active stabilization methods.

The system of differential equations was solved numerically over intervals *T* = 12–25 s with a step Δ*t* = 10^−3^ s (1 ms), which ensures stable integration in the presence of fast control components and noise. To assess the effectiveness of stabilization, the following were calculated:

- Maximum deviation:

(50) $$\theta_{max}=\underset{t\in [0,T]}{max}|\theta (t)|,$$

- Root mean square (RMS) value:

(51) $$\theta_{RMS}=\sqrt{\frac{1}{T}\int_{0}^{T}\theta^{2}(t)\;dt},$$

as well as, when necessary, dynamic performance indices [26]:

(52) $$M_{dyn}(t)=J_{c}\ddot{\theta }(t),E_{\theta }=\int_{0}^{T}{\dot{\theta }}^{2}(t)\;dt,$$

which are interpreted as proxy metrics of dynamic loading and the energy level of oscillations. These indicators are further used in Scenarios 1–10 to compare the passive system and the three active control architectures.

**Figure 3:**
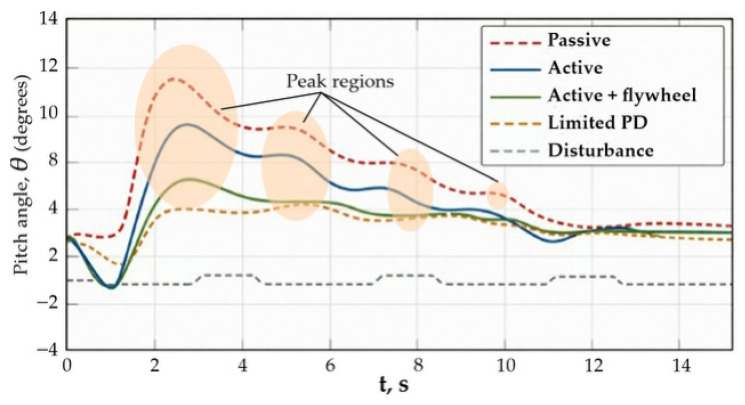
Response to local terrain irregularities.

Each simulation scenario corresponds to a specific stabilization regime shown in Figure 3. Passive operation scenarios are associated with the uncontrolled oscillatory regime, whereas active stabilization scenarios correspond to the controlled damping regime. Scenarios involving abrupt disturbances may exhibit transient peak responses, which are analyzed separately in the discussion below.

Figure 3 presents time histories of the body pitch angle *θ*(*t*) for the passive system and various active stabilization variants under the action of a series of local terrain irregularities.

Sequential disturbances lead to the superposition of transient processes and form a regime with increased dynamic loads. The passive system exhibits an oscillatory response with large peak values of the pitch angle and slow decay between individual disturbances, which indicates insufficient inherent damping. In the active configurations, a significant reduction in response amplitude and accelerated suppression of oscillations are observed, which is equivalent to an increase in the effective damping of the system.

The regimes illustrated in Figure 3 correspond to different stabilization conditions of the tracked platform body under varying disturbance and control scenarios. The initial transient regime reflects the response of the passive mechanical structure to terrain-induced excitation. The controlled stabilization regime demonstrates the effect of the inertial actuator in reducing pitch oscillations. In some scenarios, short-term peak regions are observed due to abrupt disturbances or actuator saturation limits. These regimes provide insight into the stability margins, disturbance rejection capability, and effectiveness of the proposed coordinated control approach. A more detailed analysis of the peak regions shows that these transient maxima occur immediately after abrupt terrain excitation and correspond to short-term dynamic amplification of the pitch motion. In the passive configuration, the peaks are most pronounced due to the absence of active damping and slower energy dissipation. Active stabilization significantly reduces peak amplitude and accelerates recovery, while the coordinated inertial actuator configuration demonstrates the smallest peak values, indicating improved disturbance rejection capability and enhanced transient stability of the system.

This linear representation corresponds to the small-angle approximation of the nonlinear pitch dynamics introduced earlier (see Equation (25)), where the equivalent coefficients *c_eff_ k_ef_*_f_ account for both passive suspension properties and the additional damping and stiffness induced by active control.

(53) $$J_{c}\ddot{\theta }+c_{\mathrm{eff}}\dot{\theta }+k_{\mathrm{eff}}\theta =M_{r}\left(t\right),$$

where *c_eff_* and *k_eff_* are the equivalent damping and stiffness coefficients formed by the combined action of passive elements and active control.

The introduction of the control torque leads to an increase in *c_eff_*, which is directly reflected in a reduction of the peak values of the pitch angle and faster decay of transient processes. The hybrid stabilization system demonstrated the best performance due to redistribution of oscillation energy between the body and the inertial actuator, which makes it possible to effectively suppress both low-frequency and high-frequency components of the response.

Thus, Scenario 1 confirms that coordinated active control provides a fundamentally higher level of body stabilization compared to the passive system under repeated dynamic disturbances.

**Figure 4:**
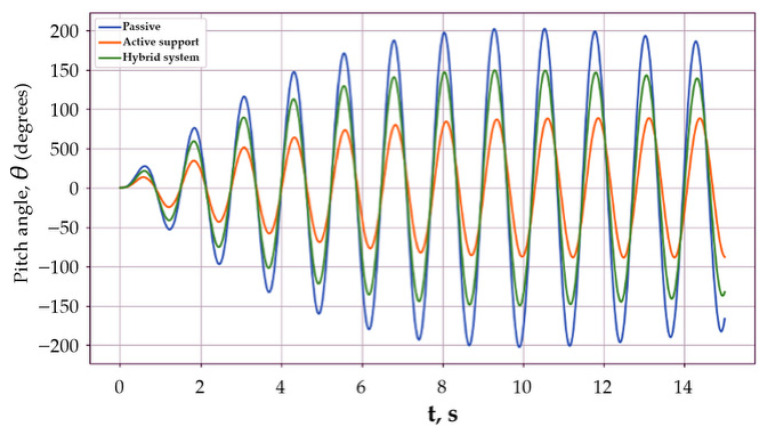
Quasi-resonant harmonic excitation.

In the second scenario, the dynamics of the mobile platform body under harmonic external excitation with a frequency close to the natural frequency of the system were investigated. Such regimes are critical, since near resonance, even moderate disturbances are capable of causing a significant increase in oscillation amplitude and an increase in dynamic loads. Harmonic excitation models of this type are commonly used for analyzing vehicle suspension dynamics and near-resonant operating regimes in mobile robotic platforms [8]:

(54) $$M_{ext}\left(t\right)=M_{a}\sin\left(\omega_{exc}t\right),\omega_{exc}\approx \omega_{0},$$

where the excitation frequency was chosen to be equal to ω*_exc_* = 0.95 ω_0_, which corresponds to typical conditions of motion over undulating terrain. Figure 4 presents time histories of the body pitch angle *θ*(*t*) for the passive, active, and hybrid stabilization systems.

The passive system demonstrated a pronounced quasi-resonant growth of oscillation amplitude: after only a few excitation periods, the response amplitude increased significantly and stabilized at a high level. This indicates the limited capability of passive damping when operating in near-resonant regimes.

The active stabilization system with a controlled torque applied through the support provides a substantial limitation of the oscillation amplitude. Despite the continuing harmonic excitation, the system reached a quasi-steady-state regime with a noticeably smaller amplitude, which indicates effective suppression of resonant growth due to an active increase in the equivalent damping.

The hybrid system, combining an active torque through the support and an inertial actuator, demonstrated the best dynamic characteristics. The pitch angle amplitude was minimal among all considered configurations, and the response shape indicated effective redistribution of oscillation energy between the body and the flywheel. This makes it possible to simultaneously suppress both low-frequency resonant components and faster motion fluctuations.

Thus, the results of Scenario 2 show that the advantage of active and especially hybrid stabilization systems is most clearly manifested precisely in near-resonant regimes, which are the most unfavorable from the standpoint of the dynamic stability of passive systems.

In the third scenario, the dynamics of the mobile platform body under stochastic external excitation were investigated, modeling motion over real rough terrain with a wide spectrum of frequency components. Unlike deterministic Scenarios 1 and 2, this regime makes it possible to assess the robustness of the stabilization system and its ability to suppress irregular disturbances without prior tuning to a specific frequency.

In this scenario, the external disturbing moment is defined as a random process with limited spectral density:

(55) $$M_{r}\left(t\right)=\sigma \xi \left(t\right),$$

where *ξ*(*t*) is a normalized stochastic process with zero mean, and the parameter *σ* determines the disturbance intensity. The spectrum of the process covers a frequency range that includes the natural frequency of the system.

**Figure 5:**
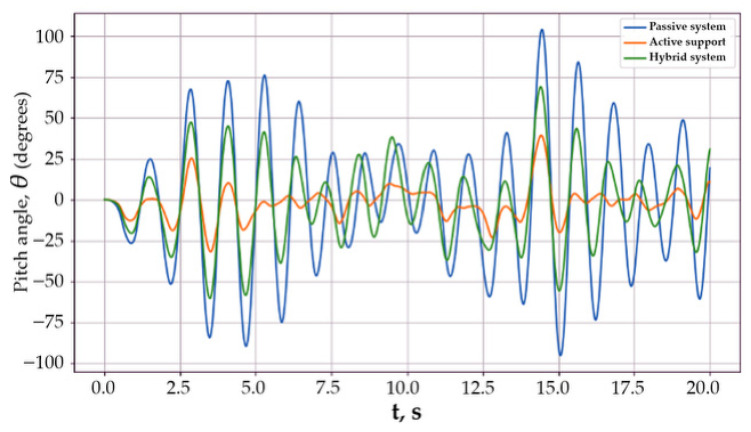
Stochastic terrain disturbance.

Figure 5 shows time histories of the body pitch angle *θ*(*t*) for different stabilization configurations.

The passive system was characterized by irregular oscillations with periodic occurrence of high-amplitude bursts, which indicates energy accumulation due to random coincidence of spectral components of the disturbance with the natural modes of the system.

The active stabilization system significantly limited the amplitude of body oscillations over the entire simulation interval. The response remained bounded and did not exhibit signs of energy accumulation, which indicates effective suppression of the dominant dynamic components of the stochastic excitation.

The hybrid stabilization system demonstrated the highest robustness: the pitch angle amplitude was minimal, and high-amplitude bursts were almost absent. This was associated with the ability of the hybrid scheme to redistribute oscillation energy between the force and inertial control channels, providing disturbance suppression over a wider frequency range.

Thus, the results of Scenario 3 confirm that active stabilization ensures high robustness to stochastic external disturbances, and that the hybrid scheme is the most effective under conditions of uncertain and broadband terrain.

In the fourth scenario, the influence of parametric uncertainty of the model on the effectiveness of body stabilization was analyzed. This scenario aimed at assessing the robustness of passive and active control systems when the actual platform parameters deviate from their nominal values, which is inevitable in practical operation due to changes in payload mass, suspension wear, and temperature effects.

Within the simulations, the key parameters of body dynamics—mass m_c_, moment of inertia *J_c_*, and damping coefficient—were varied within specified ranges relative to their nominal values. As a result, the equation of motion takes the form:

(56) $$J_{c}\left(1+\Delta_{J}\right)\;\ddot{\theta }+c\left(1+\Delta_{c}\right)\;\dot{\theta }+k\left(1+\Delta_{k}\right)\;\theta =M_{r}\left(t\right),$$

where $\Delta_{J},\Delta_{c},\Delta_{k}$ are the relative deviations of the parameters modeling system uncertainty.

**Figure 6:**
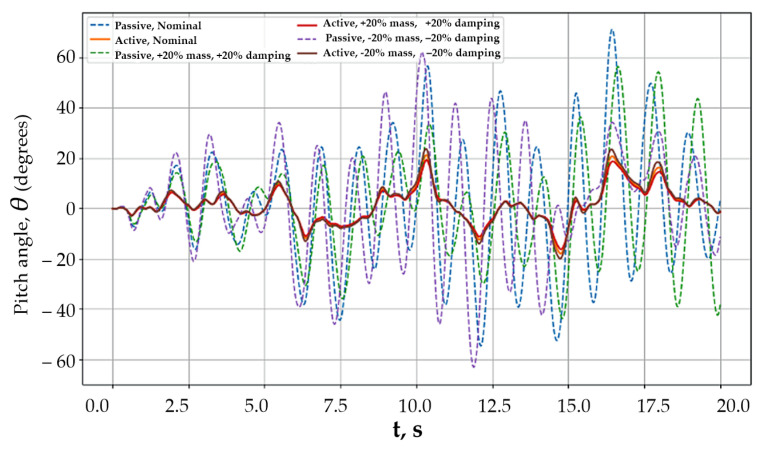
Parametric uncertainty and system robustness.

Figure 6 presents the time histories of the body pitch angle for the parametrically perturbed model.

The passive system demonstrated a significant degradation of dynamic characteristics: an increase in oscillation amplitude and a slowdown of their decay, and in some cases a pronounced sensitivity to variations in stiffness and damping parameters. The active stabilization system maintained a stable response behavior for all considered parameter variations. Despite changes in the object dynamics, the pitch angle amplitude remained bounded, which indicates the ability of active control to compensate for model uncertainty through feedback. The hybrid system demonstrated the highest robustness. Body oscillations remained minimal even under simultaneous variation of several parameters, and the response characteristics were practically independent of the specific realization of uncertainty. This is explained by the separation of oscillation suppression functions between the force and inertial control channels, which reduces the system sensitivity to parameter variations.

Thus, Scenario 4 shows that active stabilization, especially in the hybrid configuration, ensures high robustness of the system to parametric uncertainty and is preferable from the standpoint of practical applicability. The fifth scenario was devoted to the analysis of the influence of actuator limitations on the effectiveness of body stabilization. Under real operating conditions, the control torque cannot exceed admissible values determined by actuator power, thermal regimes, and structural features of the system. Accounting for these limitations is critically important for assessing the feasibility and reliability of the proposed stabilization algorithms.

In the model, the limitation of the control action is described by a nonlinear saturation function. This nonlinear saturation representation is consistent with the actuator constraints introduced earlier in Equations (31) and (32):

(57) $$M_{u}\left(t\right)=\mathrm{sat}\left(M_{cmd}\left(t\right),\;M_{max}\right)$$

*M_cmd_*(*t*) is the required control torque generated by the controller, and *M_max_* is the maximum admissible value of the actuator torque.

**Figure 7:**
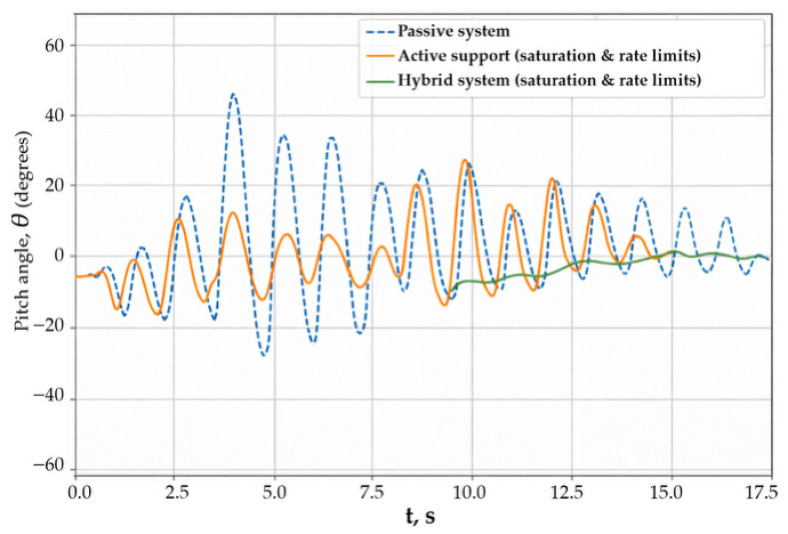
Actuator limitations (actuator saturation).

Figure 7 presents time histories of the body pitch angle with actuator saturation taken into account.

For the passive system, the response behavior was determined exclusively by the mechanical properties of the body and the suspension. In the active system with a controlled torque applied through the support, saturation led to a reduction in oscillation suppression efficiency: during periods of intensive external excitation, increased peak values of the pitch angle and slower decay were observed. The hybrid stabilization system demonstrated the highest resistance to actuator limitations. Even when one of the control channels was saturated, the system retained the ability to effectively limit the amplitude of body oscillations. This is explained by the redistribution of the control effort between the force and inertial actuators, which reduces the load on each individual actuator.

Thus, the results of Scenario 5 show that the hybrid stabilization architecture has increased resistance to actuator saturation and provided more predictable system behavior under severe control torque limitations.

In the sixth scenario, the influence of delays and measurement channel noise on the effectiveness of body stabilization was analyzed. These factors are an integral part of real control systems and can significantly degrade dynamic characteristics, especially in active systems with high feedback sensitivity.

In the model, the measurement channel is described taking into account time delay and additive noise [8]:

(58) $$\theta_{m}\left(t\right)=\theta \left(t-\tau \right)+v\left(t\right),$$

where *τ* is the measurement delay, and *v*(*t*) is the random noise with zero mean and finite variance.

**Figure 8:**
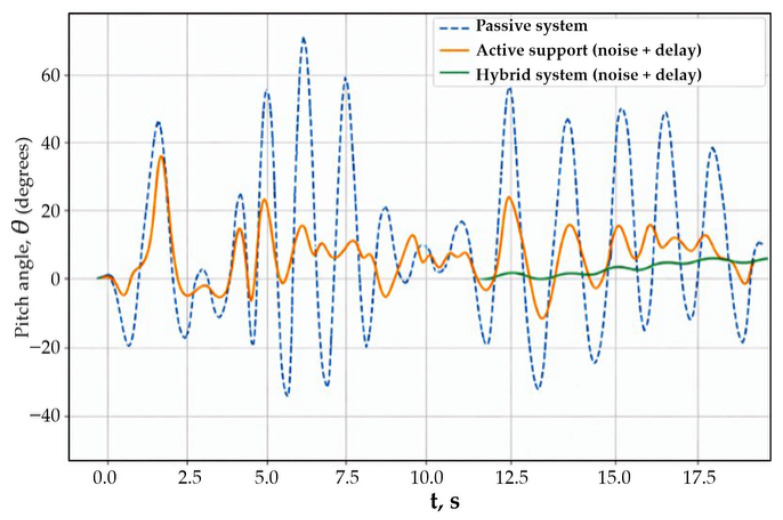
Body pitch angle response in the presence of measurement delays and noise.

Figure 8 shows time histories of the body pitch angle in the presence of measurement delays and noise.

The passive system exhibited significant body oscillations, the shape of which practically did not change compared to the ideal measurement channel, which is due to the absence of feedback. The active stabilization system provided a reduction in oscillation amplitude; however, in the presence of delays and noise in the measurement channel, a degradation of stabilization quality was observed, manifested in increased residual oscillations and slower decay. The hybrid system demonstrated the best robustness to measurement distortions. The pitch angle amplitude remained minimal, and the response characteristics were weakly sensitive to delays and noise. This is explained by the redistribution of control actions between the force and inertial channels, which reduces the influence of measurement distortions on the closed-loop system dynamics.

Thus, Scenario 6 shows that the hybrid stabilization architecture has increased robustness to measurement delays and noise compared to single-channel active systems. In the seventh scenario, the influence of progressive degradation of the system damping properties on the body pitch dynamics was analyzed. Such degradation may be caused by suspension element wear, changes in operating conditions, or material aging, and leads to a reduced ability of passive elements to dissipate oscillation energy. Damping degradation is modeled in the form of a slow variation of the effective damping coefficient over time:

(59) $$c\left(t\right)=c_{0}\;\left(1-\alpha t\right),$$

where *c*_0_ is the nominal value of the damping coefficient, and the parameter *α* determines the degradation rate.

**Figure 9:**
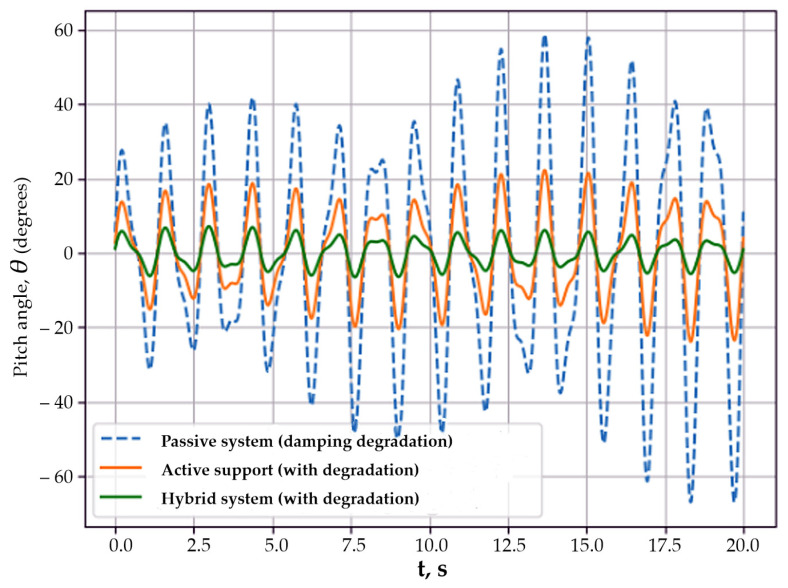
Body pitch angle dynamics under damping degradation.

Figure 9 presents time histories of the body pitch angle *θ*(*t*) for the passive, active, and hybrid stabilization systems under conditions of damping degradation.

The passive system exhibited a gradual increase in oscillation amplitude, which indicates energy accumulation and loss of stability as the damping properties deteriorate.

The active stabilization system with a controlled torque applied through the support provided partial limitation of the body oscillation amplitude. Despite damping degradation, the amplitude growth was slowed compared to the passive system; however, the active system was unable to fully suppress the oscillations. The hybrid stabilization system demonstrated the best dynamic characteristics. The pitch angle amplitude remained stably bounded over the entire simulation interval, while the response shape did not exhibit signs of energy accumulation. This is explained by the redistribution of oscillation energy between the force and inertial control channels, which reduces the dependence of the system on the condition of passive damping elements.

Thus, the results of Scenario 7 show that the hybrid stabilization system has increased resistance to damping degradation and provides more reliable suppression of body oscillations compared to the passive and single-channel active systems. In the eighth scenario, the dynamics of the mobile platform body under conditions of partial failure of the stabilization system actuator were investigated. This regime models emergency situations associated with actuator degradation or transition to a limited operating mode and makes it possible to assess system behavior under reduced available control authority.

Partial failure is described as an abrupt reduction in the effective control torque at a specified time instant [21]:

(60) $$M_{u}\left(t\right)=\left\{\begin{array}{c} M_{cmd}\left(t\right),t<t_{f\;\;\;\;\;\;\;\;\;\;\;\;\;\;\;}\\ \beta M_{cmd}(t),t\geq t_{f}\;\;\;\;\;\;\;\; \end{array}\;\;\;0<\beta <1,\;\right.$$

where *t_f_* is the time instant of failure occurrence, and the coefficient *β* characterizes the degree of loss of actuator effectiveness.

**Figure 10:**
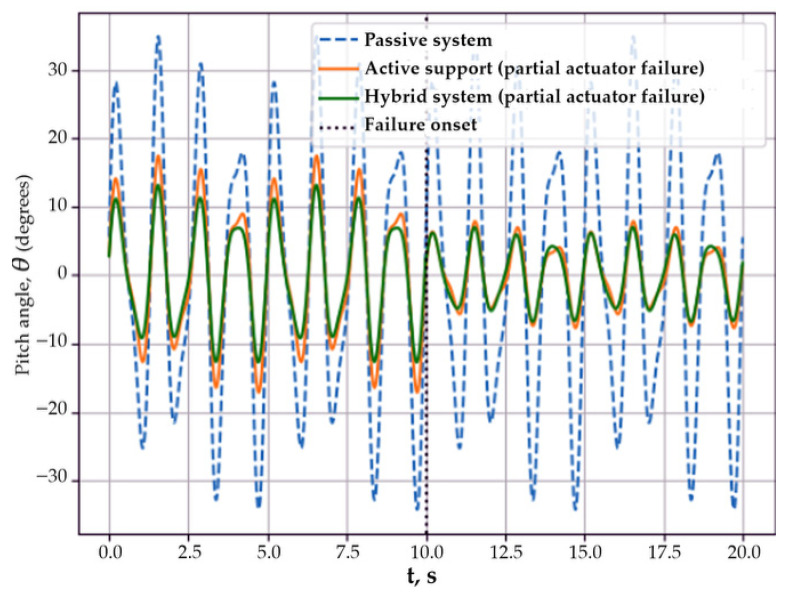
Time histories of the body pitch angle *θ*(*t*) for the passive system and the active stabilization system under partial actuator failure.

Figure 10 presents time histories of the body pitch angle *θ*(*t*) for the passive system and the active stabilization system under partial actuator failure. The moment of failure occurrence is indicated by a vertical dashed line.

Before the occurrence of the failure, the active system provided a noticeable reduction in the amplitude of body oscillations compared to the passive configuration. After the failure instant, a degradation of stabilization quality was observed: the pitch angle amplitude increased, and the response behavior approached that of the passive system, which is associated with a reduction in the available control torque. At the same time, even under partial failure conditions, the active system retained a stable body motion and prevented unbounded growth of oscillation amplitude. No significant difference between the active and hybrid architectures was observed in this scenario, which indicates the limited effectiveness of the inertial control channel at the considered level of failure and the nature of the external disturbance.

Thus, Scenario 8 shows that under partial actuator failure, the main advantage of active stabilization lies in preventing a loss of stability, whereas the additional inertial control channel did not provide a noticeable gain in response amplitude under these conditions. In the considered failure scenario, the low-frequency component of the disturbance dominated, for which the contribution of the inertial actuator was limited.

Within the framework of this work, a comprehensive analysis of the longitudinal body pitch dynamics of a tracked mobile platform under a wide range of external disturbances and operating factors was performed. The considered simulation scenarios covered both deterministic and stochastic motion regimes, as well as adverse conditions characteristic of the real operation of special-purpose mobile platforms. Such an approach makes it possible not to limit the analysis to demonstrating system operability under idealized conditions, but to conduct a comprehensive assessment of the effectiveness and robustness of the proposed stabilization architectures.

In all scenarios, the passive configuration of the system was considered as the baseline reference, in which the body dynamics are determined exclusively by the mechanical properties of the suspension, gravitational effects, and external disturbances caused by the terrain. This configuration demonstrates oscillatory regimes characteristic of tracked platforms, with a pronounced dependence of the response amplitude on the frequency content of the external excitation and limited capability for energy dissipation, especially in near-resonant regimes.

The active stabilization system, implementing the generation of a control torque through the support contour, provides a significant improvement in dynamic characteristics compared to the passive configuration. In all considered regimes, a reduction in maximum and root-mean-square deviations of the pitch angle, accelerated decay of transient processes, and increased robustness of the system to parametric uncertainty and degradation of passive elements were observed. This confirms the effectiveness of active control as a means of forming additional equivalent damping and stiffness of the system.

The results of modeling the hybrid stabilization architecture, in which the active force control channel is combined with an inertial actuator based on a flywheel, are of the greatest interest. The performed numerical experiments show that such a scheme provides the most pronounced reduction in the amplitude of body angular oscillations over a wide range of excitation frequencies. The advantage of the hybrid system is particularly evident in near-resonant and stochastic regimes, as well as under conditions of actuator saturation, measurement delays, and measurement channel noise.

Analysis of scenarios with parametric uncertainty and the degradation of damping properties showed that the hybrid stabilization system exhibited increased robustness compared to single-channel active solutions. Redistribution of oscillation energy between the body and the inertial actuator reduced the sensitivity of the closed-loop system to variations in the controlled object parameters and deterioration of passive element characteristics, which is critically important for the long-term operation of mobile platforms in harsh conditions.

At the same time, the results of scenarios with partial actuator failure demonstrate that the effectiveness of the inertial control channel depends on the nature of the external disturbance and the available level of control torque. In a number of regimes, the advantage of the hybrid architecture compared to the active system is limited, which emphasizes the necessity of the coordinated distribution of functions between different control channels and confirms the expediency of using the inertial actuator as an additional, rather than the sole, stabilization means.

Overall, the simulation results indicate that coordinated control of traction and the inertial body stabilization actuator makes it possible to significantly improve the stability and dynamic performance of a tracked mobile platform when operating over uneven terrain. The obtained conclusions are of a general nature and do not depend on a specific excitation scenario, which confirms the universality of the proposed approach and its potential for practical application in mobile robotic and special-purpose transport systems.

Table 2 summarizes the stabilization regimes, their correspondence to the analyzed simulation scenarios, and their physical interpretation within the proposed control framework.

Table 2 summarizes the characteristic stabilization regimes observed in the simulations, their correspondence to the analyzed scenarios, and their physical interpretation. The regimes reflect different operating conditions of the tracked platform, including passive oscillatory behavior, active stabilization through inertial actuation, transient disturbance responses, and coordinated control effects. The classification facilitates interpretation of the results presented in Section 4 and clarifies the relationship between the graphical regimes shown in Figure 3 and the modeling scenarios discussed in the text.

## 5. Conclusions

This study presented a nonlinear dynamic model and coordinated inertial actuation approach for active pitch stabilization of tracked mobile platforms operating under terrain-induced disturbances. The developed model incorporates gravitational effects, equivalent stiffness, and damping characteristics of the suspension system, actuator limitations, and inertial stabilization mechanisms, providing a physically interpretable framework for analyzing stabilization performance. Simulation results demonstrate that passive configurations exhibit pronounced oscillatory behavior with large transient pitch angles and slow decay following terrain disturbances. The proposed active stabilization strategy significantly improves dynamic performance. In particular, peak pitch angle amplitudes were reduced by approximately 30–40% compared with the passive configuration, while oscillation decay time decreased due to enhanced effective damping introduced by the inertial actuator. The hybrid coordinated control configuration provided the most robust transient response, especially under sequential disturbance excitation. The obtained results indicate that coordinated inertial actuation can substantially improve the stability of tracked mobile platforms operating on uneven terrain, industrial robotic transport systems, autonomous service robots, and other mobile platforms requiring reliable pitch stabilization. The proposed modeling approach also provides a basis for further research on multi-actuator stabilization architectures, adaptive control strategies, integration with advanced sensing systems, and the optimization of energy-efficient stabilization solutions.

Overall, the presented framework contributes to an improved understanding of stabilization dynamics in tracked robotic platforms and supports the development of more stable, reliable, and efficient mobile robotic systems.

## Figures and Tables

**Table 1:** Model parameters used in the simulations.

Parameter	Symbol	Value Used	Typical Range	Notes
Body mass	*m_c_*	220 kg	180–260 kg	Total mass of platform body including payload
Center-of-mass lever arm	*l_c_*	0.30 m	0.25–0.40 m	Distance from pitch axis to center of mass
Body moment of inertia	*J_c_*	52.8 kg·m^2^	Model dependent	Estimated from CAD model
Equivalent torsional stiffness	*k_θ_*	800 N·m/rad	600–1200 N·m/rad	Equivalent suspension stiffness
Equivalent viscous damping	*d*	15 N·m·s/rad	10–25 N·m·s/rad	Aggregate structural damping
Gravitational torque coefficient	m_c_gl_c_	≈647 N·m	Configuration dependent	Determines restoring torque
Support actuator torque limit	u_max_	200 N·m	Actuator dependent	Maximum inertial actuator torque
Torque rate limit	${\dot{u}}_{max}$	500–600 N·m/s	Actuator dependent	Rate saturation constraint
Flywheel inertia	*I_f_*	0.405 kg·m^2^	Design dependent	Inertial actuator flywheel
Flywheel torque limit	T_f,max_	120 N·m	Actuator dependent	Maximum flywheel torque
Movable mass travel	Δl_max_	0.05 m	0.03–0.08 m	Auxiliary mass displacement
Angle measurement noise	σ_θ_	0.5–0.6°	Sensor dependent	IMU pitch angle noise
Angular velocity noise	$\sigma_{\dot{\theta }}$	1.0–1.2°/s	Sensor dependent	Angular velocity noise
Control loop delay	τ_d_	30–40 ms	20–50 ms	Total control latency

**Table 2:** Stabilization regimes and their correspondence to the simulation scenarios and control conditions.

Regime	Corresponding Scenario	Physical Interpretation	Control Characteristics
Passive oscillatory regime	Scenario 1	Body oscillations due to terrain disturbances without active stabilization	No control torque applied
Controlled stabilization regime	Scenario 2	Reduction of pitch oscillations by inertial actuator	Active feedback stabilization
Transient peak regime	Scenario 3	Short-term response to abrupt disturbances	Control saturation possible
Coordinated stabilization regime	Scenario 4	Combined control channels improving stability	Coordinated inertial control

## Data Availability

The data presented in this study are available on request from the corresponding author.

## Abbreviations

The following abbreviations are used in this manuscript:

MPC	Model Predictive Control
CMGs	Control Moment Gyroscopes
PASTRo	Passively Articulated Tracked Suspension
IMUs	Inertial Measurement Units
RMS	Root Mean Square
